# A new parameter using serum lactate dehydrogenase and alanine aminotransferase level is useful for predicting the prognosis of patients at an early stage of acute liver injury: A retrospective study

**DOI:** 10.1186/1476-5926-7-6

**Published:** 2008-08-14

**Authors:** Kazuhiro Kotoh, Munechika Enjoji, Masaki Kato, Motoyuki Kohjima, Makoto Nakamuta, Ryoichi Takayanagi

**Affiliations:** 1 Department of Medicine and Bioregulatory Science, Graduate School of Medical Science, Kyushu University, 3-1-1 Maidashi, Higashi-ku, Fukuoka, 812-8582, Japan

## Abstract

**Background:**

Although most patients with severe acute hepatitis are conservatively cured, some progress to acute liver failure (ALF) with a high rate of mortality. Based on the evidence that over-activation of macrophages, followed by disturbance of the hepatic microcirculation, plays a key role in ALF, we hypothesized that the production of serum lactate dehydrogenase (LDH) might increase in the liver under hypoxic conditions and could be an indicator to discriminate between conservative survivors and fatal patients at an early stage.

**Results:**

To confirm this hypothesis, we developed a new parameter with serum alanine aminotransferase (ALT) and LDH: the ALT-LDH index = serum ALT/(serum LDH - median of normal LDH range). We analyzed retrospectively 33 patients suffering acute liver injury (serum ALT more than 1000 U/L or prothrombin time expressed as international normalized ratio over 1.5 at admission) and evaluated the prognostic value of the ALT-LDH index, comparing data from the first 5 days of hospitalization with the Model for End-Stage Liver Disease (MELD) score. Patients whose symptoms had appeared more than 10 days before admission were excluded from this study. Among those included, 17 were conservative survivors, 9 underwent liver transplantation (LT) and 7 died waiting for LT. We found a rapid increase in the ALT-LDH index in conservative survivors but not in fatal patients. While the prognostic sensitivity and specificity of the ALT-LDH index was low on admission, at day 3 they were superior to the results of MELD.

**Conclusion:**

ALT-LDH index was useful to predict the prognosis of the patients with acute liver injury and should be helpful to begin preparation for LT soon after admission.

## Background

Acute liver failure (ALF) or fulminant liver failure is a disease characterized by abrupt onset and high mortality. Liver transplantation (LT) is the only effective treatment for ALF and many patients die before undergoing LT because of rapid progression of the disease [1,2]. Therefore, a prompt decision regarding LT is required following an early determination of prognosis. Among the various clinical selection criteria proposed for LT, the King's College criteria and the Model for End-Stage Liver Disease (MELD) criteria have been applied widely [3,4]. However, those criteria include some factors reflecting multiple or systemic organ failure, which means that many patients fulfilling the criteria are already too unwell for transplantation to be contemplated. The poor prognosis of ALF seems to be attributable to the definition of the disease itself. Generally, ALF is defined as an acute liver disease complicated with hepatic encephalopathy and severe coagulopathy. Considerable efforts made in the past to improve the prognosis of ALF have shown limitations. It is well known that supportive methods such as plasma exchange and hemodialysis are not necessarily efficacious once encephalopathy develops in patients suffering from severe acute hepatitis [5-8]. In order to improve the overall prognosis of ALF, it is necessary to seek ways to select patients who have the possibility of developing hepatic encephalopathy before the symptom appears, rather than struggle to cure the patients after fulfilling the ALF criteria. Of course, a new strategy is required to prevent the progression of the disease.

The difficulty in predicting the prognosis of ALF is mainly attributable to incomplete elucidation of its mechanism. The most characteristic pathological finding of ALF is massive necrosis without regeneration, which implies the involvement a disturbance of the hepatic circulation in the progression of the disease. Although this idea is not novel and has not been regarded as important, we believe that it should be revisited, considering recent reports of over-activation of macrophages in the liver, which is believed to cause hepatic hypoxia as a result of disturbance of the microcirculation [9-12]. Although the importance of over-activation of hepatic macrophages in the progression of ALF may be accepted, it is difficult to demonstrate the occurrence of this phenomenon. Whilst liver biopsy is a reliable means of confirming macrophage proliferation in the liver, it carries a risk of bleeding, especially with the coagulopathy seen in ALF. Therefore, we focused on lactate dehydrogenase (LDH), which is recognized as an enzyme released in liver injury, as are aspartate aminotransferase and alanine aminotransferase (ALT). It is common to regard monitoring serum LDH as of little value because it is produced in various organs and the specificity for liver disease is low. However, in ischemic liver disease, the elevation of serum LDH is more pronounced than that of ALT [13-16]. Several pieces of evidence that the production of LDH increases in hypoxic conditions have been reported [17-19]. Another consideration regarding serum LDH in liver disease is its more rapid decline than ALT, because of its shorter half-life in serum [20]. Against the background of these findings, we hypothesized that the ALT-LDH ratio could be a marker indicating the degree of hepatic hypoxia caused by macrophage over-activation, which might be helpful to discriminate between fatal patients and conservative survivors at an early stage of ALF. In this study, we examined retrospectively the correlation between the serum ALT-LDH ratio of the patients suffering from acute liver injury and who had had the possibility of developing ALF and their outcomes, and evaluated the predictive efficacy of this new indicator compared to the MELD scoring system.

## Results

Comparing the backgrounds on admission between LT or death cases and the conservative survivors, the former were older and had a higher proportion of patients with ascites and hepatic encephalopathy (Table 1). The laboratory data on admission showed that the LT or death cases had significantly lower serum levels of albumin and longer PT. Concerning the enzyme activities in serum, the average values for AST and ALT were higher in conservative survivors, while LDH was lower, although the difference was not significant. The MELD score on admission was about 9 points higher in the LT or death cases; however, their average value was below 30, probably reflecting that the patients were at an early stage of their clinical courses.

**Table 1:** Characteristics of the patients.

	**Alive**	**LT or Death**	**Total**	**p-value**
Age	39.4 ± 15.3	48.3 ± 16.3	43.7 ± 16.2	0.0382
Sex (M/F)	10/7	8/8	18/15	0.6109
Ascites (+/-)	2/15	10/6	12/21	0.0025
Encephalopathy (+/-)	2/15	9/7	11/22	0.0067
AST (U/L)	4122.4 ± 3915.1	3587.3 ± 4123.7	3862.9 ± 3963.4	0.5523
ALT (U/L)	3845.5 ± 2932.1	2777.8 ± 2850.6	3327.8 ± 2898.5	0.2275
LDH (U/L)	2668.0 ± 3431.0	2796.1 ± 4093.2	2730.1 ± 3707.3	0.8289
ALP (U/L)	543.7 ± 168.1	509.4 ± 162.5	527.1 ± 163.7	0.9139
γ-GTP (U/L)	293.1 ± 200.0	245.8 ± 249.8	270.2 ± 223.2	0.171
Total bilirubin (mg/dL)	9.1 ± 6.8	14.6 ± 10.0	11.8 ± 8.8	0.0689
Direct bilirubin (mg/dL)	6.1 ± 4.7	9.4 ± 6.9	7.6 ± 6.0	0.1395
Albumin (g/dL)	3.7 ± 0.4	3.3 ± 0.4	3.5 ± 0.4	0.0034
PT-INR	2.19 ± 1.56	3.38 ± 2.29	2.76 ± 2.00	0.0013
Platelet (× 10^4^/μL)	14.9 ± 5.9	11.5 ± 5.9	13.3 ± 6.1	0.0717
Creatinine (mg/dL)	1.14 ± 2.03	1.35 ± 1.61	1.25 ± 1.81	0.5757
Etiology – HAV	3	3	6	0.9811
Etiology – HBV	8	5	13	0.9811
Etiology – Drug	2	1	3	0.9811
Etiology – Wilson	1	1	2	0.9811
Etiology – Unknown	3	6	9	0.9811
MELD score	17.66 ± 9.79	26.69 ± 11.89	22.0 ± 11.6	0.0059
ALT-LDH index 3.0	8	8	16	0.8658
ALT-LDH index ≥ 3.0	9	8	17	0.8658

Values were expressed by mean ± standard deviation. LT: Liver transplantation, GGT: Gamma-glutamyl transferase.

**Figure 1:**
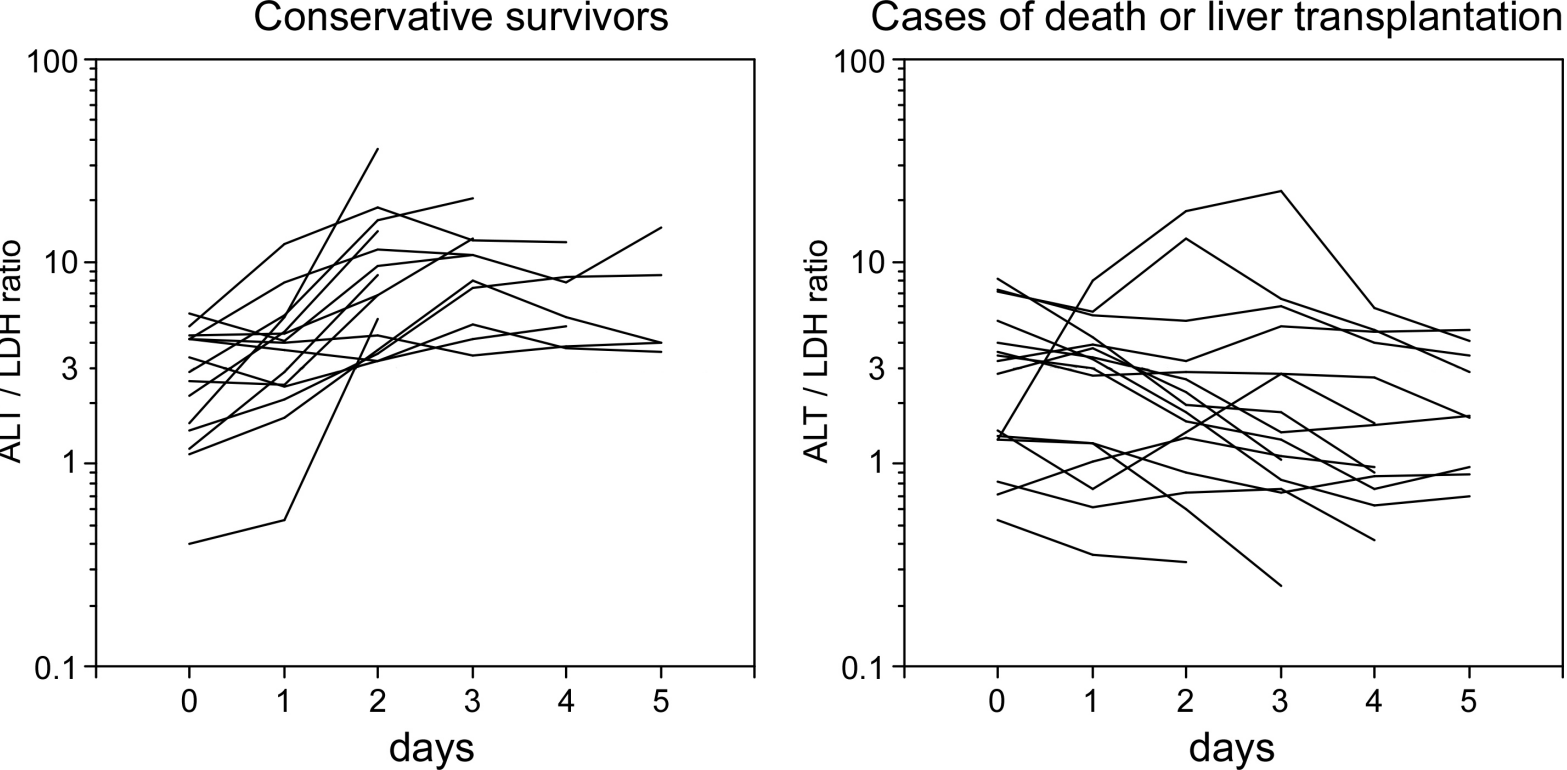
**Changes in the ALT-LDH index over the first 5 days after admission**. In most of the conservative survivors, a rapid elevation of the index was observed. Once the serum LDH activity reached the normal range (below 229 U/L), subsequent plotting was avoided. Two patients who had serum LDH within the normal range at admission are not included in the figure. On the contrary, the index decreased or remained constant in most of the patients who died before LT or underwent LT.

**Figure 2:**
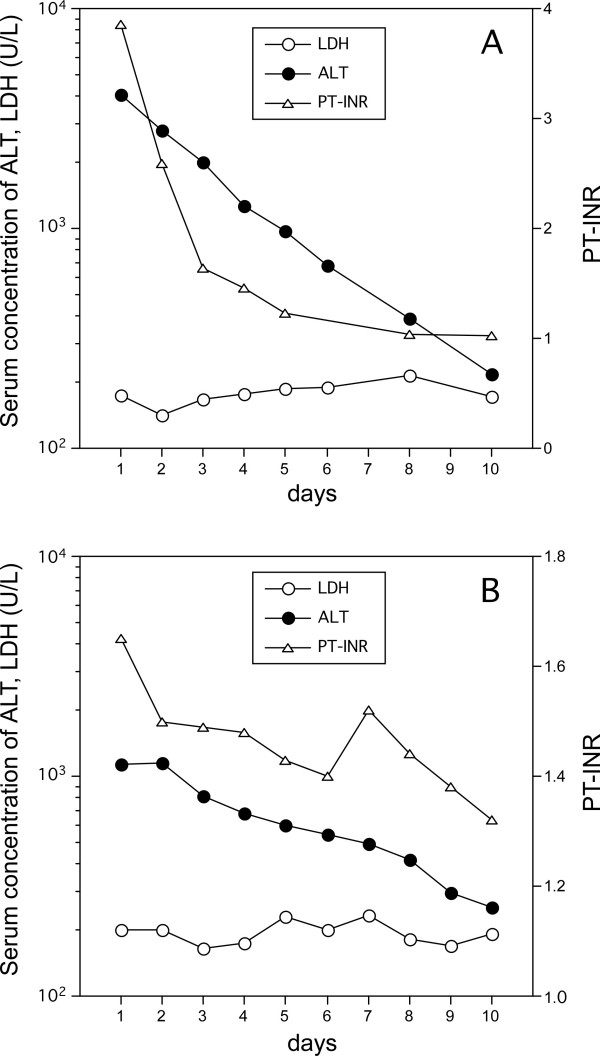
**Patients with normal serum LDH activity at admission**. The clinical courses of the two patients who had normal serum LDH activity at admission despite high levels of serum ALT. Their liver function improved rapidly without particular intervention.

The serum ALT levels on admission were over 1000 U/L in 27 patients, and decreased quickly (23 patients) or remained steady (10 patients) during the first three hospital days. This finding also indicated that the periods from the onset of the disease to admission of the patients were relatively short. There was one patient who showed re-elevation of serum ALT after the third hospital day, triggered by HBV. There was no particular tendency of ALT transition during the first five days in the conservative survivors or the LT or death cases. On the other hand, the transition of the ALT-LDH index during the same period differed between the two categories: the index increased quickly in most of the conservative survivors while it tended to remain low in the LT or death cases (Figure 1). We confirmed that there was no evidence indicating haemolysis for all enrolled patients. There were two patients with normal serum LDH but high serum ALT activity on admission, both belonged to the conservative survivors group. As shown in Figure 2, both showed rapid improvement of serum ALT and PT-INR after hospitalization, without any particular support.

**Figure 3:**
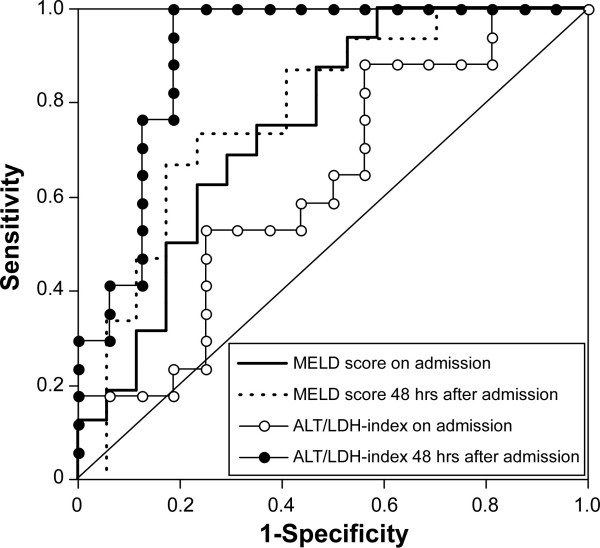
**ROCs using the MELD score or ALT-LDH-index**. The curves from the MELD score for the first and third hospital days are similar. On the other hand, the ALT-LDH index on the third day improved in sensitivity and specificity compared with the curve for the day of admission.

The ROC curves predicting conservative survivors are illustrated with the ALT-LDH index and MELD score using data from the first and the third hospital days, respectively (Figure 3). The MELD score showed similar curves using the first and third days' data, while the ALT-LDH index for the third day showed much higher sensitivity and specificity, and was superior to the MELD score, although the curve for the first day was close to the identity line corresponding to a complete lack of discriminative power. The area under the ROC curve of the ALT-LDH index for the third day was 0.893 while that of the MELD score was 0.777 (Table 2).

**Table 2:** ROC curves with MELD score and ALT-LDH index predicting conservative survivors.

	**Area under ROC**	**Std. Error**	**95% C.I.**	**p-value**
MELD (day1)	0.750	0.0857	0.582 – 0.918	0.0143
MELD (day3)	0.777	0.0841	0.612 – 0.941	0.00779
ALT-LDH (day1)	0.574	0.102	0.373 – 0.774	0.471
ALT-LDH (day3)	0.893	0.0629	0.770 – 1.02	0.000118

In past reports evaluating the predictive efficacy of the MELD score for ALF, the cut-off line was set between 30 and 35. As shown in Table 3, the MELD score from the first day data showed a high specificity of 88.24%, but a low sensitivity of 31.25%, at cut-off line 30 and this tendency did not change at cut-off line 35. On the contrary, both the sensitivity and specificity calculated by the ALT-LDH index with a cut-off of 3.0 increased from the first day to the third day: 75% and 100%, respectively.

**Table 3:** Prognostic values of MELD score and ALT-LDH index predicting conservative survivors.

	**Sensitivity (%)**	**Specificity (%)**	**PPV (%)**	**NPV (%)**	**Efficiency (%)**
MELD (day1) <30	31.3	88.2	71.4	57.7	60.6
MELD (day1) <35	18.8	94.1	75	55.2	60.6
MELD (day3) <30	33.3	88.2	71.4	60	62.5
MELD (day3) <35	20	94.1	75	57.1	59.4
ALT-LDH (day1) >3.0	50	52.9	50	52.9	51.5
ALT-LDH (day3) >3.0	75	100	100	81.0	87.9

PPV: positive predictive value, NPV: negative predictive value.

## Discussion

In this study we demonstrated the contrasting transitions of the ALT-LDH index in the early stage of acute liver injury between the conservative survivors and the patients with progressive fatal liver failure. In the former, the ALT-LDH index increased abruptly soon after the peak of serum ALT elevation, which was caused by a more rapid decrease of LDH than ALT activity. This phenomenon is convincing because the half-life of serum LDH is normally much shorter than that of serum ALT. On the other hand, in the fatal patients group, a less rapid decrease of serum LDH activity kept the ALT-LDH index low, which implied that the delayed decrease of serum LDH at an early stage of ALF may be closely related to a poor prognosis. This phenomenon might be explained by assuming hypoxic conditions in the livers of the patients with progressive ALF.

Although the mechanism of ALF has not been elucidated fully, several authors recently reported that over-activation of macrophages plays a key role in the progression of ALF [9-12]. The activated and proliferating macrophages in the liver could injure endothelial cells and cause a disturbance in the hepatic microcirculation. We suppose that this may be the main process of ALF, at least for the non-acetaminophen type. Meanwhile, LDH is an essential enzyme involved in anaerobic glycolysis and is responsible for the anaerobic transformation of pyruvate to lactate. Increased expression of LDH under hypoxic conditions has been demonstrated in various cell lines [17-19]. Concerning liver diseases, it is well known that dominant elevation of serum LDH is observed in hypoxic hepatitis caused by shock or heart failure [13-16]. Although the elevation of LDH activity in acute liver injury has been simply supposed to be enzyme leakage through damaged hepatocyte membranes, as the seen with ALT, increased LDH production could also be attributable to anaerobic conditions. The hepatocytes are expected to increase the production of LDH under anaerobic conditions, until they become necrotic. From this viewpoint, the persistent low ALT-LDH index in fatal patients might be the result of increased production of LDH from residual living hepatocytes in hypoxia. Prolonged hypoxic conditions could cause massive or lobular necrosis, which coincides with the pathologic findings of ALF.

When we accept the mechanism described above, acute liver injury could be supposed to consist of two different processes of cell destruction. One is direct cytotoxicity toward hepatocytes caused by various triggers. In most non-acetaminophen hepatitis, cytotoxic T cells attack hepatocytes directly. In this process, the increased release of enzymes into the serum is the result of simple leakage from injured hepatocytes, and enzyme activities decrease rapidly, according to their half-lives, as soon as the triggers are removed or inactivated. The other mechanism is hypoxic liver injury caused by disturbance of the hepatic microcirculation. The persistent low ALT-LDH index may imply the situation that the hypoxic process mainly remains after the removal of the trigger of liver injury. Most acute liver injury might be explained as a mixture of these two mechanisms, to various degrees. The patients shown in Figure 3 are supposed to be representatives of cases that almost lack a hypoxic process.

In the past, many attempts have been made to predict the prognosis of ALF [21-24]. However, it is impossible to estimate the prognosis using data from a single time point at a very early stage because ALF is a disease with rapid progression and patients may present at various phases of the clinical course. The MELD score is certainly useful to predict the prognosis of patients awaiting LT. However, as shown by our results, its sensitivity remained very low over several days after admission. It is a matter of course because the MELD score was determined principally using data from patients in their end stage. On the other hand, while the sensitivity and specificity of the ALT-LDH index were rather poor on admission, both were improved dramatically beyond the MELD score at day 3. That is, the ALT-LDH index could reflect the rapid clinical change of ALF. We emphasize that the important thing is to observe the transition of clinical data, not simply a single time-point, in a disease with rapid progression, such as ALF.

## Conclusion

In this study, we showed the efficacy of the ALT-LDH index to predict the prognosis of patients with acute liver injury at their early stages. This index should enable us to begin preparation for LT shortly after admission. We believe that the index could be a support for other indicators, such as the MELD score. However, the number of the enrolled patients into this study was not enough. The further evaluation in larger prospective clinical studies is required.

## Methods

### Patients

Patients with severe acute liver injury referred to our hospital for consideration for LT between April 2000 and March 2004 were analyzed retrospectively. Among them, those with serum ALT activity more than 1000 U/L or prothrombin time expressed as international normalized ratio (PT-INR) over 1.5 were enrolled into this study, amounting to 33 patients (Table 1). In order to focus on the early phase of ALF, those in whom the onset of any of clinical symptoms, such as general fatigue, appetite loss, nausea and jaundice, had begun 10 days before admission were excluded from this study. Hepatic encephalopathy grade 2 or more was seen in 11 (33%) on admission. The etiology of liver injury varied: 6 hepatitis A virus (HAV), 13 hepatitis B virus (HBV), 3 drugs other than acetaminophen, 2 Wilson's disease and 9 indeterminate. Laboratory data were checked daily in the morning. Plasma exchange was performed in the afternoon when hepatic encephalopathy was greater than grade 2 or prolonged downhill PT activity was observed. Among the enrolled patients, 17 were conservative survivors, 9 underwent LT and 7 died waiting for LT. In following analysis we considered the fatal patients and those who were transplanted as one category because the pathological examination showed that the livers of all transplant recipients were markedly atrophic and entirely necrotized, which indicated that they would have not been able to survive without LT.

### ALT-LDH index

The serum ALT and LDH activities were measured using the 7500 Clinical Analyzer (Hitachi High-Technologies Corporation, Tokyo, Japan). LDH was assayed using an enzymatic rate method with lactate as the substrate (lactate-pyruvate direction). ALT assay was performed without pyridoxal phosphate supplementation. The normal ranges of ALT and LDH were 6–30 U/L and 119–229 U/L, respectively. We aimed to evaluate the increase of these enzymes above normal levels and developed a new index calculated by following formula:

ALT-LDH index = serum ALT/(serum LDH - median of normal LDH range)

In acute liver injury, both serum ALT and LDH commonly decrease after the peak observed in the acute phase regardless of the prognosis. However, in the patients with fatal prognosis, the decrease of serum LDH is expected to delay compared with that of serum ALT, which would be caused by microcirculation disturbance in liver. Therefore, if we use the serum LDH value as a predictive marker of ALF, it should be evaluated under connection with the serum ALT value. Although the simple ALT/LDH ratio seems to be acceptable in evaluation of the serum activity of LDH connecting to ALT, it could not reflect the degree of those enzymes' elevation from normal level when they are in relatively low levels because of the difference of their normal ranges. On the other hand, the value of ALT-LDH index distinctly increases when the serum LDH decreases close to the normal range.

For the enrolled patients, this index was calculated for the first 5 days from their admission, comparing the changes in serum ALT activity during the same period. According to the normal range of our assay system, the median of the serum LDH was calculated as 174 U/L in this study.

### Statistical analysis

Differences in clinical backgrounds and laboratory data between conservative survivors and fatal patients, including those who underwent LT, were analyzed using the Χ2-test and Student t-test. The utilities of the ALT-LDH index and MELD score were evaluated using receiver operating characteristic (ROC) curves. The sensitivity, specificity, positive and negative predictive values (PPV and NPV), efficiency, and area under the ROC curve were calculated for each indicator.

## Competing interests

The authors declare that they have no competing interests.

## Authors' contributions

KK conceived the design of the study and prepared the manuscript. ME and MK participated in the study design. MK analyzed clinical data. MN and RT drafted the paper. All authors read and approved the final manuscript.
